# LEF-1 Regulates *Tyrosinase* Gene Transcription *In Vitro*

**DOI:** 10.1371/journal.pone.0143142

**Published:** 2015-11-18

**Authors:** Xueping Wang, Yalan Liu, Hongsheng Chen, Lingyun Mei, Chufeng He, Lu Jiang, Zhijie Niu, Jie Sun, Hunjin Luo, Jiada Li, Yong Feng

**Affiliations:** 1 Department of Otolaryngology Head and Neck Surgery, Xiangya Hospital, Central South University, Changsha, Hunan, People’s Republic of China; 2 Province Key Laboratory of Otolaryngology Critical Disease, Xiangya Hospital, Central South University, Changsha, Hunan, People’s Republic of China; 3 State Key Laboratory of Medical Genetics, Central South University, Changsha, Hunan, People’s Republic of China; 4 Department of Otolaryngology, 1st Affiliated Hospital, Xinjiang Medical University, Urumqi, People’s Republic of China; University of Alabama at Birmingham, UNITED STATES

## Abstract

*TYR*, *DCT* and *MITF* are three important genes involved in maintaining the mature phenotype and producing melanin; they therefore participate in neural crest cell development into melanocytes. Previous studies have revealed that the Wnt signaling factor lymphoid enhancer-binding factor (LEF-1) can enhance *DCT* and *MITF* gene expression. However, whether LEF-1 also affects *TYR* gene expression remains unclear. In the present study, we found that LEF-1 regulated *TYR* transcription *in vitro*. LEF-1 overexpression increased *TYR* gene promoter activity, whereas LEF-1 knockdown by RNA interference significantly decreased *TYR* expression. Moreover, the core GTTTGAT sequence (-56 to -50) within the *TYR* promoter is essential for the effect of LEF-1 on TYR expression, and chromatin immunoprecipitation (ChIP) assay indicated that endogenous LEF-1 interacts with the *TYR* promoter. In addition, we observed a synergistic transactivation of the *TYR* promoter by LEF-1 and MITF. These data suggest that Wnt signaling plays an important role in regulating melanocyte development and differentiation.

## Introduction

Melanocytes and their progenitor cells melanoblasts originate from multi-potential neural crest stem cells, which then migrate through the developing embryo and localize to specific sites in the body. In addition, they comprise a stem cell pool for their regeneration [1,2], which may be an important factor in melanoma development from melanocytes [3]. The visible pigmentation in skin, hair and eyes primarily depends on the presence of melanin, a macromolecule synthesized by melanocytes. Tyrosinase is considered the key enzyme in melanogenesis initiation, as normal melanin formation does not occur without tyrosinase, and the lack of this enzyme causes albinism [4–8]. Tyrosinase is encoded by the *TYR* gene, which maps to chromosome 11q14-21 in humans and chromosome 7 in mouse [9]. Meanwhile, different types of melanin are associated with pigmentation. The TYRP1 and TYRP2/DCT proteins, with ~40% amino acid homology with TYR, have been demonstrated to play important roles in controlling the type of melanin [10–14]. Several cis-acting elements mediate the expression of these genes. For instance, microphthalmia-associated transcription factor (MITF) is a basic helix-loop-helix transcription factor, and it has been suggested to be a key regulator of *TYR* and *DCT* transcription through the E-box (CANNTG) in their promoters [15,16].

Lymphoid enhancer-binding factor 1 (LEF-1) is a member of the LEF/T-cell-specific factor (TCF) family of the high mobility group domain transcription factors, and it is a downstream nuclear Wnt signaling pathway mediator [17]. It is well established that LEF-1 is involved in the development and malignant progression of human cancers, such as melanoma, colorectal cancer, acute myeloid leukemia and pancreatic cancer [18–22]. Moreover, LEF-1 participates in embryogenesis and postnatal development by interacting with β-catenin [23–26]. LEF-1 contains three functional domains: β-catenin binding domain, context-dependent domain and high-mobility group protein domain (HMG) [17,27,28]. LEF-1 binds to the CCTTTGWW (W, A/C/T) consensus sequence in the minor groove of DNA via its HMG domain and induces a sharp bend in the DNA helix [17]. It has been shown that LEF-1 transactivates melanocyte-specific *MITF* isoform (*MITF-M*) by physically interacting with the *MITF-M* promoter [29]. It has also been shown that LEF-1 and MITF-M synergism is responsible for the regulation of *DCT* gene transcription [29].

In this study, we investigated the role of LEF-1 in the regulation of *TYR* gene expression. Our data indicated that LEF-1 binds to the *TYR* promoter and activates *TYR* gene expression. Additionally, we observed synergistic transactivation of the *TYR* promoter by LEF-1 and MITF.

## Materials and Methods

### Ethics statement

This study was approved by the Expert Committee of Xiangya Hospital of Central South University (equivalent to an Institutional Review Board). All experiments were conducted in cell lines.

### Reporter and expression plasmid construction

The luciferase reporter construct containing the human *TYR* promoter (-300 bp to +80 bp from the transcription start site) and human pCMV-3×Flag-MITF plasmid were generated as described previously [30]. The luciferase reporter constructs containing various fragments of the human *TYR* promoter (*TYR1*-Luc (-187 bp to +80 bp), *TYR2*-Luc (-23 bp to +80 bp), *TYR3*-Luc (-46 bp to +80 bp), *TYR4*-Luc (-90 bp to +80 bp)) were generated by inserting the corresponding DNA fragments into the pGL3-basic vector. A luciferase reporter construct containing the human *TYR* promoter with a mutation in the LEF-1 binding sites (*TYR5*-Luc) and with a mutation in the MITF binding sites (*TYR6*-Luc) were generated using the QuikChange II Site-Directed Mutagenesis Kit (GE Healthcare, Chalfont St. Giles, Buckinghamshire, UK)). The pcDNA3.1-LEF-1-HA expression vector containing full-length human LEF-1 cDNA (GenBank Accession No: NM_016269.4) or a dominant-negative LEF-1 (DNLEF-1) lacking the β-catenin-binding domain (amino acid residue 2–37) [27] was generated by the Nanjing Genescript Biotechnology Company (China). All plasmids were confirmed by automatic sequencing analysis.

### Cell culture, transfectionand luciferase reporter assays

Human embryonic kidney 293T (HEK293T) cells [31], human cervical carcinoma HeLa cells [32] and NIH3T3 cells [30,31] were kindly provided by JD Li (State Key Laboratory of Medical Genetics of China). Human melanoma UACC903 cell lines were purchased from the University of Arizona Cancer Center [33]. All cells were cultured in DMEM/HIGH GLUCOSE (Hyclone, Logan, UT, USA) supplemented with 10% fetal bovine serum (FBS) (Invitrogen, Carlsbad, CA, USA) at 37°C in 5% CO_2_. For some experiments, 293T cells were cultured in two different types of culture medium: serum-free DMEM and RPMI 1640 (Hyclone, Logan, UT, USA) supplemented with 10% FBS and 2 mM L-glutamine. All of the above media were supplemented with 100 U/ml penicillin/streptomycin (Invitrogen, Carlsbad, CA, USA). Transient transfection assays were conducted using Lipofectamine 2000 (Invitrogen) according to the manufacturer’s instructions. Cells were seeded at 50% confluence in 24-well plates 24 h before transfection. Cells were transfected with 5 ng reporter plasmids, 20 ng expression vector and 5 ng pCMV-β-gal (BD Bioscience/ Clontech, Palo Alto, CA, USA). The final DNA concentration added to each well was adjusted to 200 ng with empty vectors. Cells were washed with PBS and lysed with Reporter Lysis Buffer at 48 h after transfection (Promega, Madison, WI, USA). Extracts were used to determine luciferase and β-galactosidase activity. A luciferase reporter assay was conducted using the Luciferase Assay System (Promega, Madison, WI, USA) according to the manufacturer’s protocol. Luciferase activity was determined using a SIRIUS luminometer (Berthold Detection Systems, GmbH. Pforzheim, Germany) and normalized by measuring β-galactosidase activity. The relative luciferase activities were shown as the ratio of each normalized luciferase activity to the value obtained with pGL3-TYR-Luc and empty vector.

### Small interfering RNA of LEF-1 and MITF knockdown

SiRNAs specifically targeting the human *LEF-1* gene (si*LEF-1*) or *MITF* gene (si*MITF*) were designed and synthesized by RiboBioCo. Ltd. (Guangzhou, China) as follows: si*LEF-1*: (Lsi1: 5’- GGAA AAGAUCU U CGCCGAGdTdT-3’ (sense) and 5’-CUCGGCGAAGAUCUUUUCCdTdT-3’ (antisense); Lsi2: 5’-GC AAGAGACAAUUAUGGUAdTdT-3’ (sense) and 5’-UACCAUAAUUGUCUCUUGCdTdT-3’ (antisense); Lsi3: 5’-GAAAGGAGCAGGAGCCAAAdTdT- 3’ (sense) and 5’-UUUGGCUCCUGCUCCUUUCdTdT -3’ (antisense)), and si*MITF*: (Msi1: 5‘ GUACCUUUCUACCACUUUAdTdT 3‘ (sense) and 3‘ dTdTC AUGG AAAGAUGGUGAAAU 5‘ (antisense); Msi2: 5‘ GCUUGCCAUGUCCAAACCAdTdT 3‘(sense) and 3‘ dTdTCGAACGGUACAGGUUUGGU 5’; Msi3: 5‘GGCUAUGCUUACGCUUAACdTdT 3‘ and 3‘ dT dTCCGAUACGAAUGCGAAUUG 5‘). The negative control siRNA was purchased from Guangzhou RiboBioCo. Ltd. UACC903 cells were transfected with negative control siRNA (100 nM) or target gene siRNA (si*LEF-1* or si*MITF*) (100 nM) using RiboFect CP Transfection Kit (166 T) (RiboBioCo. Ltd. Guangzhou, China) according to the manufacturer’s instructions. After transfection for 72 h, cells were subjected to either Western blotting or reverse transcription polymerase chain reaction (RT-PCR).

### Reverse transcription polymerase chain reaction (RT-PCR)

Total RNA was extracted from cells using TRIzol reagent (Invitrogen, Carlsbad, CA, USA) according to the manufacturer’s instructions. A total of 2 μg RNA was used for reverse transcription with RevertAid First Strand cDNA Synthesis Kit (Thermo Fisher Scientific, NY, USA) according to the manufacturer’s protocol. Real-time PCR was performed using SYBR Premix Ex Taqll (TakaRa, Japan). The primers against *LEF-1* were: 5’-acagatcaccccacctcttg-3’ (Forward) and 5’-tgatgggaaaacctggacat-3’ (Reverse). The primers against *MITF* were: 5’-gggagctcacagcgtgtatt-3’ (Forward) and 5’-atggttcgttccttccagcg-3’. The primers against *β-actin* were: 5’-CCCATCTATGAGGGTTACGC-3’ (Forward) and 5’-TTTAATGTCACGCACGATTTC-3’ (Reverse). Primers were designed and synthesized by Sangon Biotecl Company (Shanghai, China). β-actin was used as an internal reference for normalizing mRNA expression of target genes. The PCR cycling conditions were as follows: 95°C for 5 min, followed by 40 cycles at 94°C for 30 s, 60°C for 30 s and 72°C for 30 s. The PCR reaction was performed using an iCycler IQ Multicolor Reverse-Transcription Detection System (Bio-Rad systems, Hercules, CA, USA).The relative expression of target genes was calculated by the 2^-△△ct^ method [34]. The significant differences were analyzed by SPSS 19.0 software.

### Western blots

UACC903 cells in 6-well plates were transfected with 100 μM si*LEF-1* or negative control siRNA using a RiboFect CP Transfection Kit (166 T) (RiboBioCo. Ltd., Guangzhou, China) according to the manufacturer’s instructions. Cells were lysed in 2× SDS loading buffer containing 1 mM phenylmethanesulfonyl fluoride (PMSF) (Sigma, St. Louis, WA, USA), 0.2 mM β-mercaptoethanol and protease inhibitor cocktail (Sigma)72 h after transfection. A total of 10 μg protein was separated on 10% sodium dodecyl sulfate polyacrylamide gels (SDS-PAGE), which was then transferred onto a polyvinylidene fluoride (PVDF) membrane (Millipore, Billerica, MA, USA). The membrane was blocked in Tris-buffered saline supplemented with 5% non-fat milk for 2 h at room temperature and then incubated overnight at 4°C with ChIP Ab+ LEF-1 mouse monoclonal antibody (1:1000 dilution, Millipore, Billerica, MA, USA 17604), anti-MITF mouse monoclonal antibody (1:1000 dilution, Sigma, St. Louis, WA, USA M6065), anti-TYR mouse monoclonal antibody (1:1000 dilution, Cell Signaling Technology, Boston, MA, USA 9416s) or anti-GAPDH antibody (1:5000 dilution, Abcam, Cambridge, UK, ab8245). After washing in Tris-buffered saline plus 0.1% Tween 20, the membrane was incubated with a horseradish peroxidase-conjugated secondary anti-mouse IgG antibody (1:10000, Sigma, St. Louis, WA, USA) for 1 h at room temperature. Detection was performed using the ECL plus Western blotting detection system (GE Healthcare, UK) according to the manufacturer’s instructions.

### Chromatin Immunoprecipitation (ChIP) assays

UACC903 cells were cross-linked with 1% formaldehyde and incubated at 37°C for 10 min. ChIP assay was performed according to the Millipore Biotechnology protocol with minor modifications. Cell lysates were sonicated by Covaris S2 (USA). Equal aliquots of isolated chromatin were subjected to immunoprecipitation with a ChIP Ab+ LEF-1 mouse monoclonal antibody (1:200 Millipore, Billerica, MA, USA 17604) or normal mouse IgG (Sigma, St. Louis, WA, USA) as a negative control. The immunoprecipitated DNA was used to PCR to amplify the *TYR* promoter sequence. The primers used were as follows: forward, 5’-TAACTGGGT TTGCTTAGGT-3’; reverse, 5’ -TAATACCACTCCCACCTCC-3’. The PCR products were subjected to 2% agarose gel electrophoresis.

### Co-Immunoprecipitation Assays

HeLa cells were seeded in a 100-mm plate. When cells reached 80% confluence, they were transfected with 6 μg pcDNA3.1-LEF-1-HA and 6 μg pCMV-3×Flag-MITF or its mutants using Lipofectamine 2000 (Invitrogen, Carlsbad, CA, USA), according to the manufacturer’s instructions. At 36 to 48 h after transfection, cells were lysed in RIPA buffer [150 mM NaCl, 50 mM Tris-His (pH, 8.0), 1% NonidetP-40, 2 mM EDTA, 10% Glycerol] with protease inhibitor cocktail and 1 mM PMSF. Cell lysis buffer (30 μl) was extracted and supplemented with 30 μl 2× SDS lysis buffer/0.2 mM β-mercaptoethanol for immunoblotting. The remaining lysis buffer was divided into two equal parts. Two parts were adjusted to a total volume of 1 ml with RIPA buffer, one of which was incubated with 1 μg anti-Flag M2 monoclonal antibody (Sigma, St. Louis, WA, USA, F1804) for IP with rotation overnight at 4°C, and the other aliquot was incubated with 1 μg normal mouse IgG as a negative control. A total of 30 μl protein G agarose beads were added to the samples, and the samples were incubated for another 2 h at 4°C with nutation. The beads were then washed five times in1 ml ice-cold PBS and were resuspended in 2× SDS lysis buffer/0.2 mM β-mercaptoethanol. Samples were boiled for 10 min and subjected to 12% SDS-PAGE gel electrophoresis. Immunoblot analysis was performed using an anti-HA rabbit polyclonal antibody (1:1000, Cell Signaling Technology, Boston, MA, USA 3724s).

### Statistics analysis

Data are presented as the mean ± standard deviation (SD) and were analyzed using GraphPad Prism version 5.0 software (GraphPad Software Inc. San Diego, CA, USA) unless noted otherwise. The statistical difference between two groups was analyzed using a Student’s t-test. Comparisons between more than two groups were analyzed using one-way ANOVA. A p-value less than 0.05 was considered statistically significant. All experiments were performed at least three times in triplicate on different days using different batches of cells.

## Results and Discussion

### LEF-1 knockdown decreased *TYR* expression

Wnt signaling plays a critical role in regulating melanocyte development and differentiation. It has been demonstrated that Wnt3a can increase melanin synthesis and upregulate MITF expression and its downstream target genes, including *TYR*, *DCT* and *TYRP1* [35]. Moreover, Wnt signaling inhibition suppresses melanin synthesis and MITF and TYR expression [36,37]. Wnt1- or Wnt3a-deficient mutant mice are almost devoid of pigment cells [38]. In addition, Wnt signaling activated target gene expression through the interaction of β-catenin and a LEF-1/TCF transcription factor family member [39,40].

**Fig 1:**
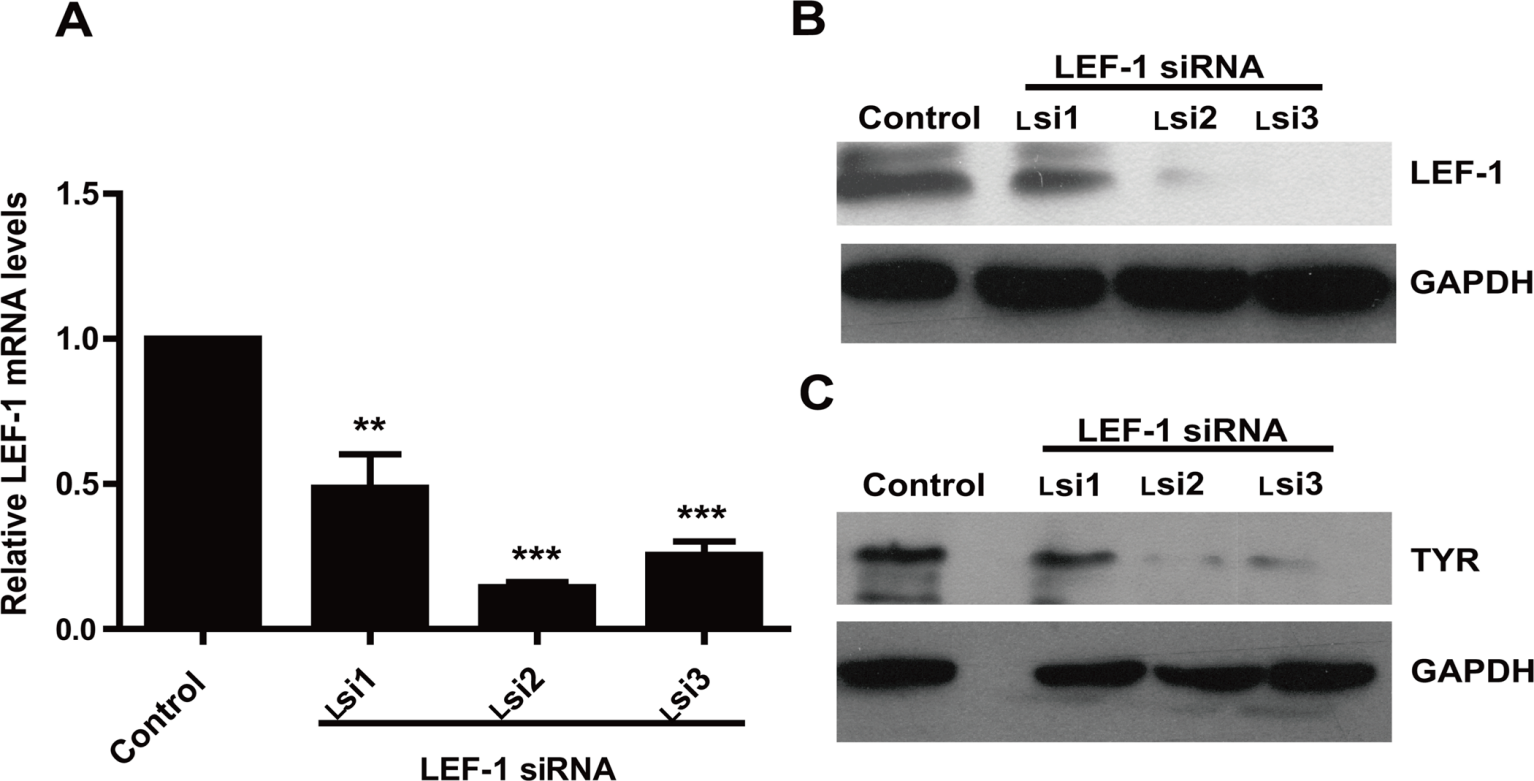
LEF-1 mRNA and LEF-1 and TYR protein levels in *LEF-1* specific siRNA-transfected UACC903 cells. **A**, RT-qPCR data showed that transfection with *LEF-1* specific siRNAs led to a significant decrease in LEF-1 expression compared to the control group, especially Lsi1 and Lsi2. An asterisk indicated statistical significance (**p<0.01, ***p<0.001 by unpaired t-test). **B, C** Western blotting was performed to examine LEF-1 and TYR protein levels in each group using a ChIP Ab+ LEF-1 mouse monoclonal antibody or anti-TYR mouse monoclonal antibody, respectively. GAPDH was used as an internal reference.

To investigate whether LEF-1 endogenously affected *TYR* gene expression, we down-regulated LEF-1 expression by using small interference RNA (siRNA). As indicated in Fig 1A, Lsi1, Lsi2 and Lsi3 each effectively decreased *LEF-1* mRNA levels. However, only Lsi2 and Lsi3 significantly down-regulated LEF-1 protein levels (Fig 1B). Moreover, we showed that *LEF-1* knockdown led to a significant decrease in TYR protein levels (Fig 1C).

### LEF-1 enhanced the human *TYR* promoter activity

**Fig 2:**
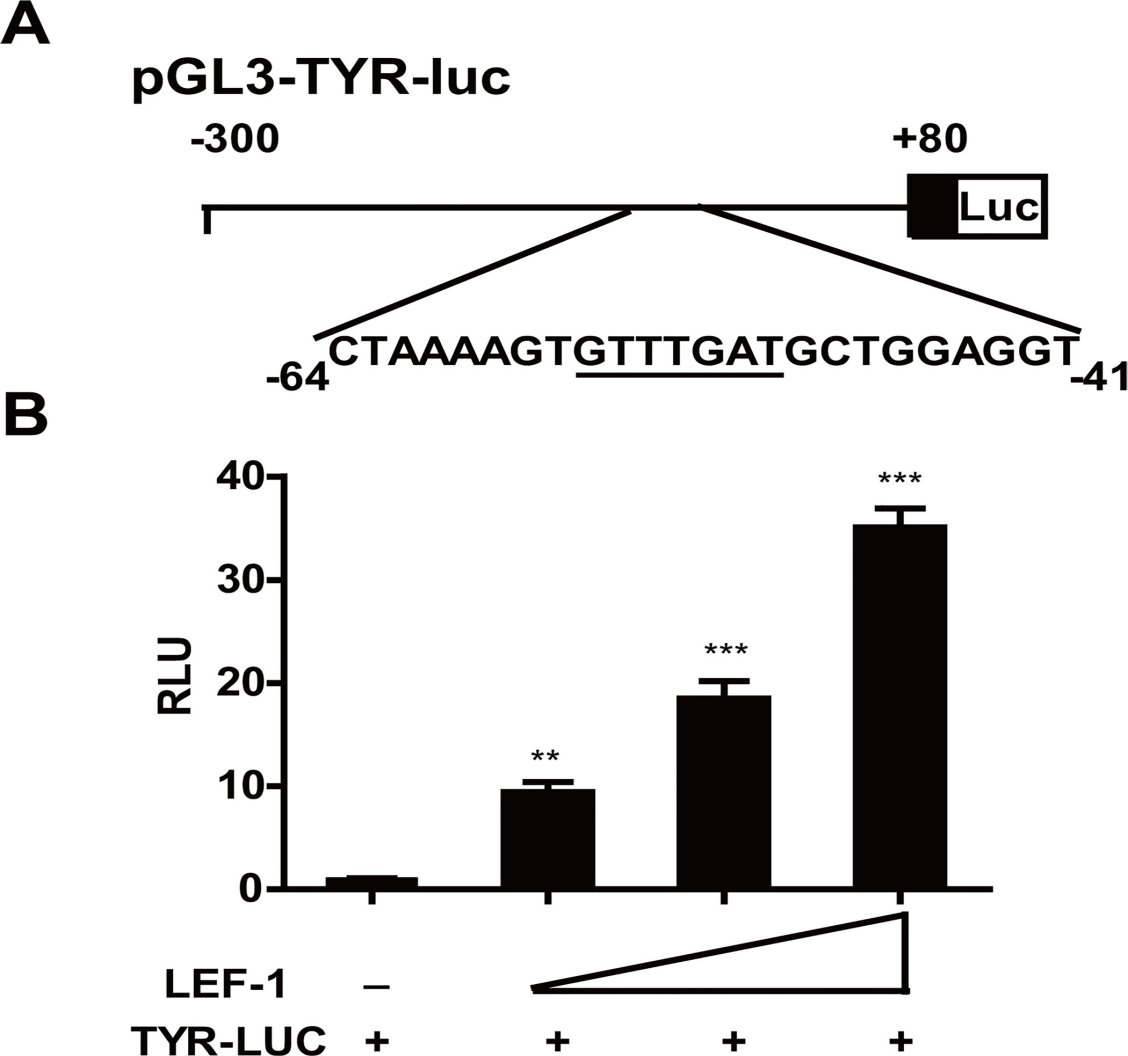
*TYR* is a direct LEF-1 target gene. **A** Schematic representation of the human TYR promoter sequence containing 380 bp (-300 bp to +80 bp) together with the potential LEF-1 binding sites (double underlined) and their flanking sequences. **B** TYR transcriptional activity determined by Luciferase activity assay. The *TYR*-Luc luciferase reporter plasmid was transiently transfected into melanoma UACC903 cells in combination with increasing amounts of LEF-1 expression vector. The basal luciferase level was set to 1. Data from all other transfections are presented as fold induction above this level. Luciferase activity was normalized by measuring β-galactosidase activity. Each value represents the mean ± SD of three replicates from a single assay. The results shown were representative of at least three independent experiments. (**p<0.01, ***p<0.001 compared to basal activity, unpaired Student’s t-test).

Because LEF-1 has been demonstrated to enhance MITF expression, which can in turn activate TYR expression [29,30,41], we examined whether LEF-1 directly or indirectly affected TYR expression, we employed the web-based bioinformatics analysis program Genomatrix browser (http://www.genomatix.de/) and identified several LEF-1 binding sites in the *TYR* promoter, suggesting that LEF-1 may directly promote *TYR* transcription. We performed a luciferase assay with a luciferase reporter construct containing -300 bp to 80 bp of the *TYR* promoter (Fig 2A). As shown in Fig 2B, LEF-1 dramatically increased *TYR* promoter activity in a dose-dependent manner. We also observed a LEF-1-induced increase in *TYR* promoter activity in several other cell lines, including HEK293T, HeLa and NIH3T3 cells (S1A Fig). Because DMEM itself and calf serum can upregulate tyrosinase or melanogenesis [42–44], we repeated the luciferase assay using serum-free DMEM or RPMI medium in UACC903 cells. As shown in S1B Fig and S1C Fig, TYR expression mildly decreased compared to its expression in DMEM medium, but the trend was similar.

**Fig 3:**
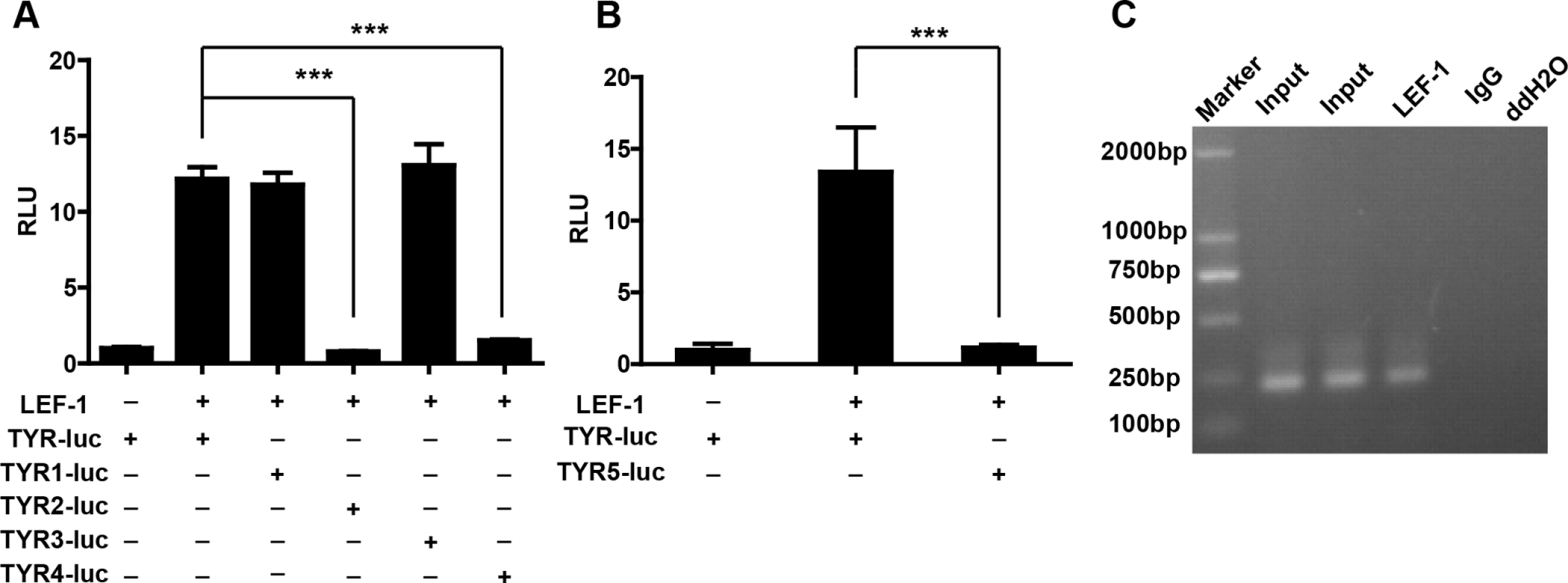
The proximal region (positions -56 bp to -50 bp) of the *TYR* promoter was required for LEF-1-mediated gene expression of the *TYR* promoter. **A, B** Transcriptional activity of LEF-1 on various *TYR* deleted or mutated promoters (*TYR5*) by luciferase assay. UACC903 cells were transiently transfected with each reporter plasmid (5 ng) and the LEF-1 expression plasmid (20 ng). Cells were lysed and luciferase activity was detected 48 h after transfection. (***p<0.001 by one-way ANOVA with Dunnett’s multiple comparison tests compared to basal activity; ^###^p<0.001; ns, not significant compared to the value from the *TYR* promoter and LEF-1 by an unpaired Student’s t-test). **C** Binding of LEF-1 to the *TYR* promoter was analyzed by ChIP assay by precipitating with the indicated antibodies and normal mouse IgG, which was used as the negative control. The crosslinks were reversed at 65°C for 4 h and digested with proteinase K (Sigma-Aldrich) for 1 h at 45°C to remove proteins. A 230-bp *TYR* promoter fragment (-28 to -257 bp) was amplified by PCR, and the products were subjected to 2% agarose gel electrophoresis. Ten percent of the chromatin DNA used for immunoprecipitation was subjected to PCR and is indicated as ‘input’. The results were representative of at least three independent experiments.

To localize the cis-acting region within the *TYR* promoter required for LEF-1-induced activation, we generated a series of reporter constructs containing a truncated *TYR* promoter. As indicated in Fig 3A, deletion of the fragment from -300 bp to -188 bp (*TYR1*-Luc) did not affect LEF-1-induced *TYR* promoter activity; however, LEF-1 failed to activate the *TYR* promoter containing only the fragment from -23 bp to + 80 bp (*TYR2*-Luc). In addition, LEF-1 activated the *TYR4*-Luc construct (-90 bp to +80 bp), but not the *TYR3*-Luc construct (-44 bp to +80 bp), suggesting that there was a LEF-1- interacting site in the region from -90 bp to -44 bp.

The region from -90 bp to -44 bp contained one putative LEF-1 binding site (AAGTGTTTGATGCTG, -60 bp to -46 bp). Accordingly, we generated a *TYR5*-Luc construct in which the core sequence GTTTGAT (-56 bp to -50 bp) was mutated to GAAAAGA. As indicated in Fig 3B, LEF-1 failed to activate *TYR5*-Luc, indicating that the core GTTTGAT sequence within the *TYR* promoter is a *bona fide* LEF-1 binding site.

We further performed a ChIP assay to confirm whether endogenous LEF-1 binds to the *TYR* promoter. We designed a primer pair containing the *TYR* promoter region from -28 bp to -257 bp. As shown in Fig 3C, the LEF-1 antibody, but not the control IgG, immunoprecipitated the DNA fragment containing the *TYR* promoter. Based on these findings, we demonstrated that LEF-1 could transactivate the *TYR* gene by binding to a conserved site in the *TYR* promoter region from -60 bp to -46 bp.

### β-catenin is required for LEF-1 to activate the TYR promoter

**Fig 4:**
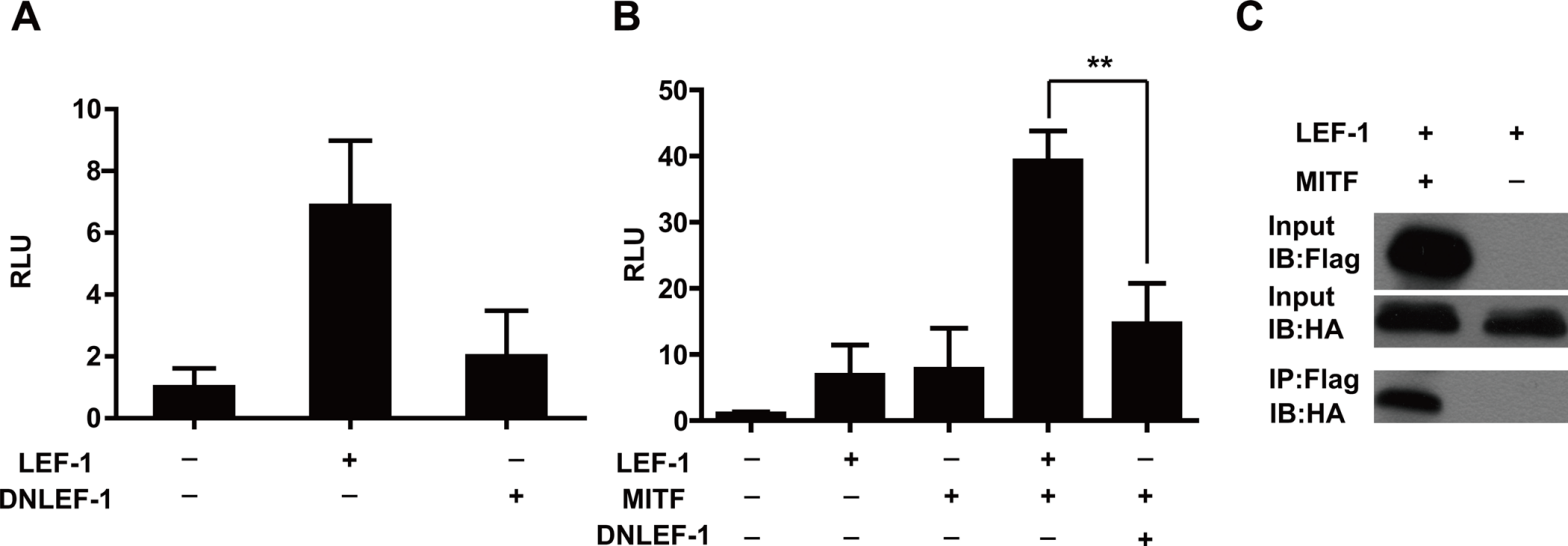
*TYR* promoter transactivation by MITF and LEF-1. **A, B** Synergism between MITF and LEF-1 and the effect of β-catenin on *TYR* promoter determined by luciferase activity assay. UACC903 cells were transfected with the *TYR* promoter-reporter plasmid and the indicated effector plasmid(s). A luciferase assay was then conducted. Relative luciferase activities are expressed as the mean ± SD from three independent experiments each performed in triplicate. (**p<0.01 by unpaired t-test).**C** Interaction between LEF-1 and MITF. Flag-tagged full-length MITF (MITF-Flag) was expressed in UACC903 cells and immunoprecipitated by the anti-Flag M2 antibody and Protein G agarose. The interaction between HA-tagged-full-length LEF-1 (LEF-1-HA) and MITF-Flag was examined by Western blotting using an anti-HA rabbit polyclonal antibody in the Flag-IP sample. MITF-Flag and LEF-1-HA protein levels were assessed by Western blotting using the corresponding antibodies.

β-catenin is a key downstream effector of the Wnt signaling pathway and functions as a transcriptional co-activator by interacting with TCF/LEF transcription factors [27,45]. Previous studies have shown that β-catenin is involved in LEF-1-mediated *MITF-M* gene expression [29,46]. Therefore, we studied whether β-catenin was also required for *TYR* promoter enhancement by LEF-1. We performed a luciferase assay with the *TYR*-Luc reporter construct and a dominant negative LEF-1 (DNLEF-1) that lacks the β-catenin-binding sites (2–37 amino acid). As shown in Fig 4A, *TYR* promoter transactivation by DNLEF-1 was dramatically reduced compared to wild type LEF-1, indicating that β-catenin is indeed required for *TYR* gene expression.

These findings indicated that LEF-1 played an important role in keeping the transcription of *TYR* gene when even lacking of Wnt signaling.

### *TYR* promoter transactivation by MITF and LEF-1

It has been reported that LEF-1 can interact with MITF-M to enhance *DCT* and *MITF-M* gene transcription [29,46]. Thus, we transfected MITF and LEF-1 with the *TYR*-Luc reporter construct into HeLa cells, which lack endogenous LEF-1 and MITF-M expression [29,47,48]. As shown in Fig 4B, we observed a synergistic transactivation of the *TYR* promoter by MITF and LEF-1. As expected, LEF-1 and MITF could be co-immunoprecipitated when co-expressed in HeLa cells (Fig 4C). Nevertheless, transfection with MITF and DNLEF-1 lacking β-catenin-binding sites led to an obvious decrease in *TYR* promoter transactivation by cooperation between MITF and LEF-1 (Fig 4B). These finding indicated that β-catenin is required for the efficient cooperation of LEF-1 with MITF-M on the *TYR* promoter.

**Fig 5:**
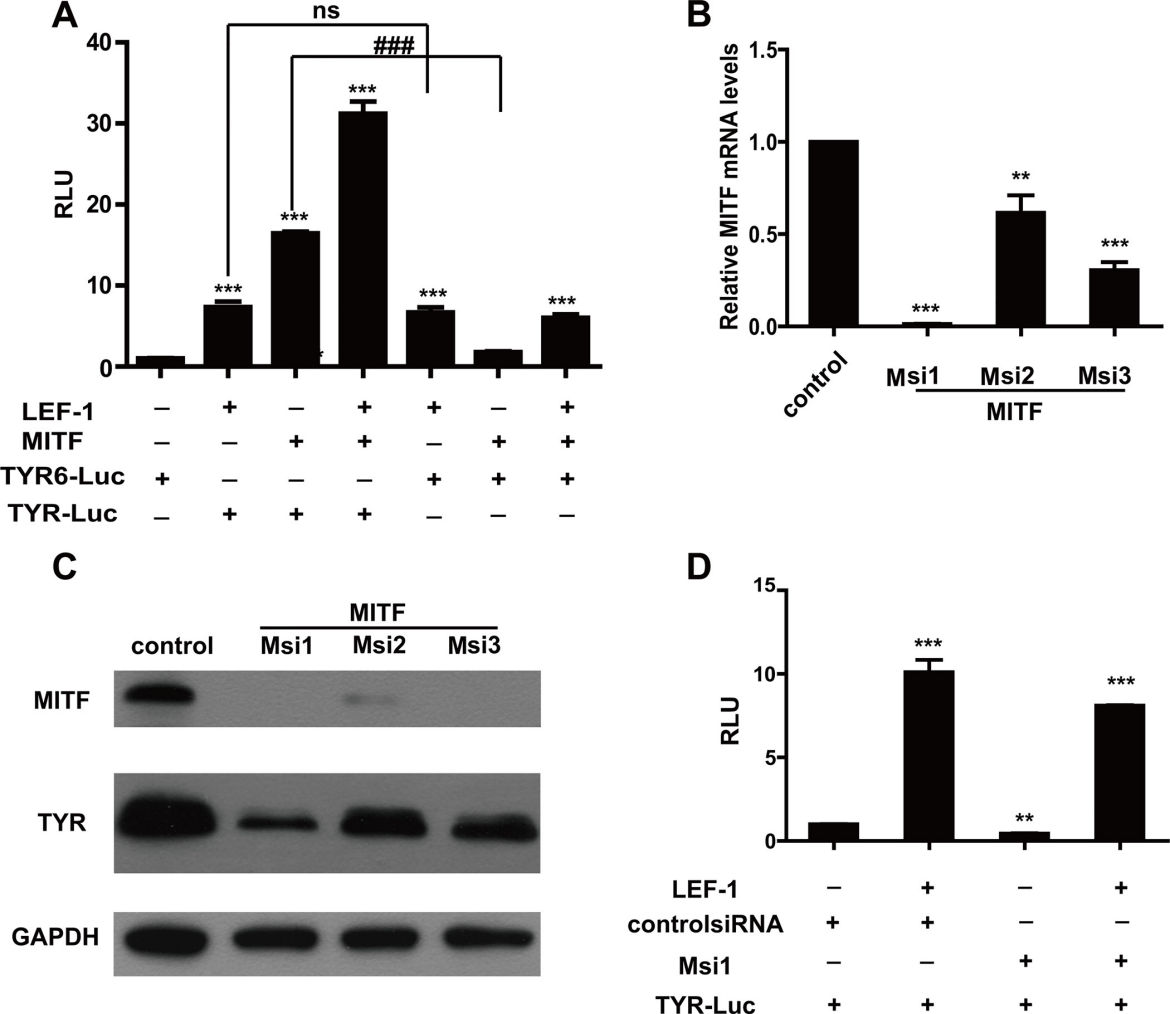
LEF-1 independently regulates *TYR* expression. **A** LEF-1-mediated tyrosinase promoter activity determined by luciferase activity assay when MITF expression was inhibited. UACC903 cells were co-transfected with the indicated promoter reporter plasmid and effector plasmid (s). A luciferase assay was performed 48 h after transfection. Relative luciferase activities are expressed as the mean ± SD from three independent experiments each performed in triplicate. (***p<0.001, compared to the value from the TYR6 promoter and empty vector; ns, not significant, ###p<0.001, compared to WT; unpaired Student’s t-test). **B, C** Examination of MITF-specific siRNAs. Melanoma UACC903 cells were transfected with MITF-specific siRNAs. RT-qPCR data showed a significant decrease in MITF expression compared to the control group, especially Msi1. An asterisk indicated statistical significance (**p<0.01, ***p<0.001 by an unpaired t-test) (B).Western blotting was performed to examine MITF and TYR protein levels in each group using an anti-MITF mouse monoclonal antibody or anti-TYR mouse monoclonal antibody, respectively. GAPDH was used as an internal reference (**C**). **D** LEF-1 significantly enhanced tyrosinase promoter activity in the presence of *MITF* siRNA. UACC903 cells were transiently transfected by the *TYR* promoter reporter in combination with LEF-1 or Msi1.Cells were lysed and luciferase assays were conducted 48 h after transfection. Basal luciferase activitywas set to 1. Data from all other transfections are presented as fold induction above basal levels. Luciferase activity was normalized by measuring β-galactosidase activity. Each value shown was the mean ± SD of three replicates from a single assay. The results shown were representative of at least three independent experiments. (**p<0.01, ***p<0.001 compared with basal activity, unpaired Student’s t-test).

MITF is considered a key transcription factor for melanocyte differentiation, and it controls *TYR* expression [30,41]. MITF transcriptionally regulates *TYR*, and LEF-1 regulates *MITF* [29]. To further demonstrate the direct effect of LEF-1 on tyrosinase transcription, we conducted a luciferase assay using a *TYR*-Luc reporter with the core MITF binding sequence CATGTG mutated to CATTTG (*TYR6*-luc). As shown in Fig 5A, MITF itself transactivated *TYR-*Luc but not *TYR6*-Luc; however, LEF-1 activated both promoters. Furthermore, we transfected cells with *MITF*-specific siRNAs, which effectively decreased *MITF* mRNA and protein levels (Fig 5B and 5C). Although *MITF* knockdown mildly decreased basal luciferase activity, LEF-1 significantly enhanced tyrosinase promoter activity in the presence of *MITF* siRNA (Fig 5D). Our results demonstrated that LEF-1 directly bound to and enhanced tyrosinase promoter activity.

In summary, our study demonstrates that LEF-1can positively mediate TYR expression. Moreover, LEF-1 is involved in the transcriptional regulation of *DCT* and *MITF-M* [29,46]. In addition, *TYR*, *DCT* and *MITF-M* are all regulated by synergism between MITF and LEF-1, and β-catenin is required for their efficient expression [29,46]. Accordingly, our study highlights an important role of Wnt signaling in the melanocyte development and differentiation.

## Supporting Information

S1 Fig *TYR* promoter activities determined by luciferase activity assay in different cells or medium. **A** Luciferase activity was detected when LEF-1 and the TYR promoter (5 ng) were co-transfected into HEK293T, HeLa or NIH3T3 cells. The basal level of all luciferase activity from the three cells was set to 1. Data from other transfections are presented as fold induction above the basal level. Luciferase activity was normalized by measuring β-galactosidase activity. The data are presented as the mean ± SD from three independent experiments each performed in triplicate. (**p<0.01, ***p<0.001 by one-way ANOVA with Dunnett’s multiple comparison tests). **B, C** Luciferase assays were repeated when LEF-1 was co-transfected with the *TYR* promoter (5 ng) into 293T cells in RPMI or serum-free DMEM cultures.
(TIF)
