# Supplementary figures and images for: LEF-1 Regulates Tyrosinase Gene Transcription In Vitro
Source: PLoS One. 2015 Nov 18;10(11):e0143142. doi: 10.1371/journal.pone.0143142 (PMC4651308; doi:10.1371/journal.pone.0143142)

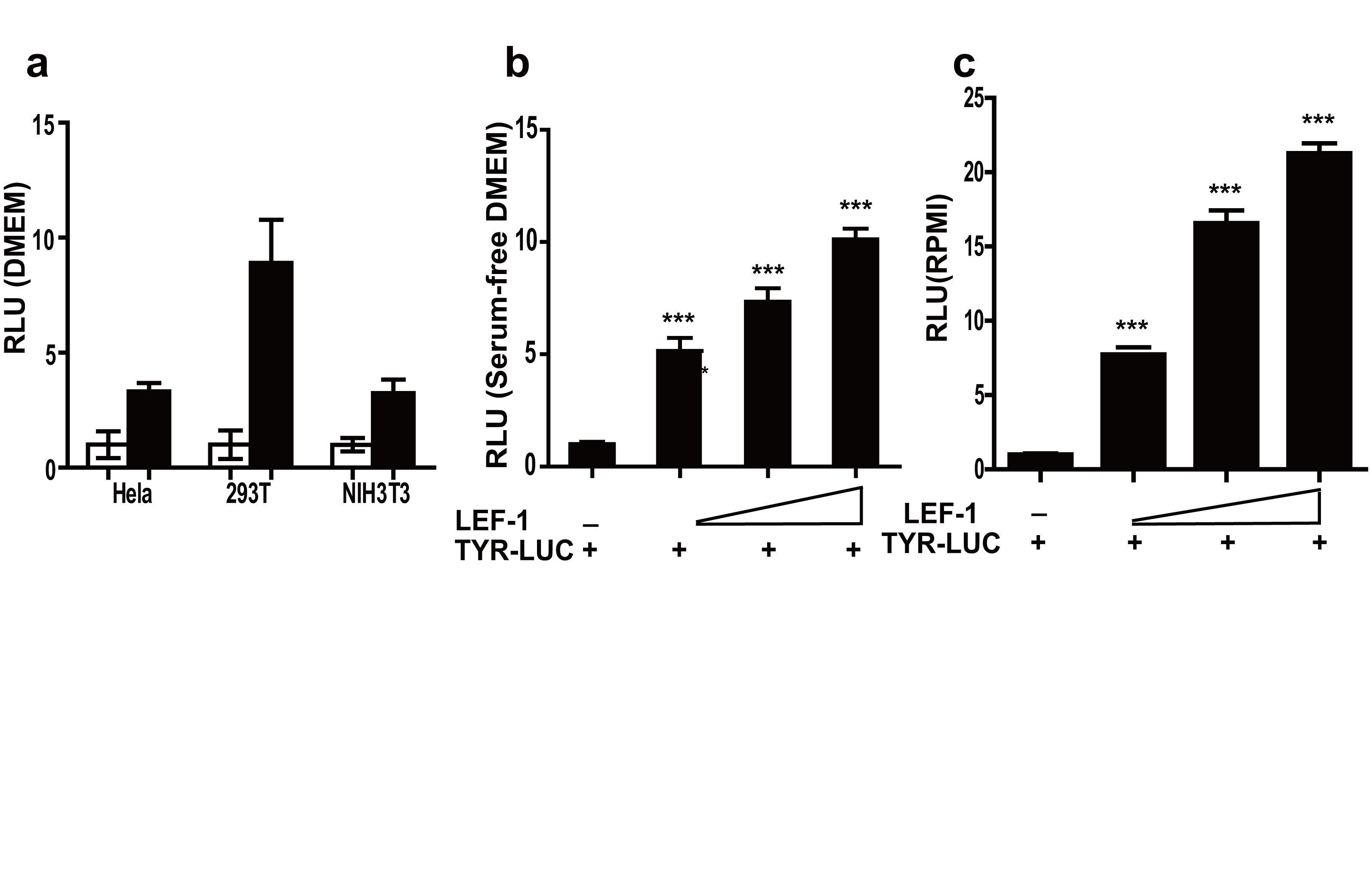

Supplement: S1 Fig — A Luciferase activity was detected when LEF-1 and the TYR promoter (5 ng) were co-transfected into HEK293T, HeLa or NIH3T3 cells. The basal level of all luciferase activity from the three cells was set to 1. Data from other transfections are presented as fold induction above the basal level. Luciferase activity was normalized by measuring β-galactosidase activity. The data are presented as the mean ± SD from three independent experiments each performed in triplicate. (**p<0.01, ***p<0.001 by one-way ANOVA with Dunnett’s multiple comparison tests). B, C Luciferase assays were repeated when LEF-1 was co-transfected with the TYR promoter (5 ng) into 293T cells in RPMI or serum-free DMEM cultures. (TIF) [file pone.0143142.s001.tif]
